# Syntax-Guided Content-Adaptive Transform for Image Compression

**DOI:** 10.3390/s24165439

**Published:** 2024-08-22

**Authors:** Yunhui Shi, Liping Ye, Jin Wang, Lilong Wang, Hui Hu, Baocai Yin, Nam Ling

**Affiliations:** 1 Faculty of Information Technology, Beijing University of Technology, Beijing 100124, China; syhzm@bjut.edu.cn (Y.S.); yeliping199903@163.com (L.Y.); wangll@emails.bjut.edu.cn (L.W.); huh@emails.bjut.edu.cn (H.H.); ybc@bjut.edu.cn (B.Y.); 2 Department of Computer Science and Engineering, Santa Clara University, Santa Clara, CA 95053, USA; nling@scu.edu

**Keywords:** image compression, adaptive compression, deep learning

## Abstract

The surge in image data has significantly increased the pressure on storage and transmission, posing new challenges for image compression technology. The structural texture of an image implies its statistical characteristics, which is effective for image encoding and decoding. Consequently, content-adaptive compression methods based on learning can better capture the content attributes of images, thereby enhancing encoding performance. However, learned image compression methods do not comprehensively account for both the global and local correlations among the pixels within an image. Moreover, they are constrained by rate-distortion optimization, which prevents the attainment of a compact representation of image attributes. To address these issues, we propose a syntax-guided content-adaptive transform framework that efficiently captures image attributes and enhances encoding efficiency. Firstly, we propose a syntax-refined side information module that fully leverages syntax and side information to guide the adaptive transformation of image attributes. Moreover, to more thoroughly exploit the global and local correlations in image space, we designed global–local modules, local–global modules, and upsampling/downsampling modules in codecs, further eliminating local and global redundancies. The experimental findings indicate that our proposed syntax-guided content-adaptive image compression model successfully adapts to the diverse complexities of different images, which enhances the efficiency of image compression. Concurrently, the method proposed has demonstrated outstanding performance across three benchmark datasets.

## 1. Introduction

The widespread use of smartphones, surveillance equipment, and social media platforms as well as potential future applications such as holographic imaging have generated a massive volume of image data. This not only poses significant demands on storage and transmission infrastructures but also drives the development of image compression technology, making it a key tool to address this challenge. The primary goal of image compression technology is minimizing data size while maintaining the visual integrity and high quality of images. Currently, there are various content-adaptive holographic compression techniques, including intra-prediction [1], histogram analysis [2], maximum phase depth utilization [3], consideration of object size [4], a vector lifting scheme [5], a quincunx lifting scheme [6], local feature determination [7], spatial–temporal analysis [8], and spatial segmentation [9]. Moreover, the application scope of image compression technology is continually expanding, including fields such as augmented reality [10,11] and 3D imaging [12,13]. With the continuous advancement in technology, image compression will continue to play an important role in improving data transmission efficiency and reducing storage costs.

The classic image compression frameworks, including JPEG [14], JPEG2000 [15], BPG [16], WebP [17], and VVC [18], incorporate key modules such as transform, quantization, and entropy encoding. Each module relies on manually designed operations working in concert to convert raw image data into a more compact form suitable for storage and transmission. Rate-Distortion Optimization (RDO) theory [19] is employed to select the coding mode, aiming to find the optimal solution with the least distortion among various mode combinations. However, under the RDO theory, seeking the optimal coding strategy is an extremely challenging optimization task. Traditional coding techniques require meticulous optimization of each module individually followed by their integration, in the hope of achieving globally optimal performance. However, this approach fails to realize joint optimization between modules, leaving room for improvement in image coding performance. Furthermore, traditional coding algorithms primarily rely on linear orthogonal transforms, such as the Discrete Cosine Transform (DCT). Related studies indicate that natural images still exhibit a significant amount of high-dimensional correlation redundancy among their features after linear transforms [20]. By employing nonlinear transforms, this redundancy can be more effectively eliminated, and the use of higher-order nonlinear transform methods is expected to further enhance coding performance [21].

The significant advancement in computational power and deep learning technology has given rise to various efficient deep learning-based image coding techniques [22,23,24,25,26,27,28]. In 2016, Ballé et al. [29] introduced the first end-to-end image coding method based on convolutional autoencoders. During the training phase, by introducing uniform noise to replace the quantization operation, they made the entire framework differentiable. Furthermore, they proposed Generalized Divisive Normalization (GDN), a method with image decorrelation properties, which has been integrated into a compression framework based on convolutional neural networks (CNNs) [30]. This framework has emerged as a mainstream approach for end-to-end image compression. In the testing phase, the input image is processed through an analysis transform network to extract features, which are then quantized and entropy encoded to generate a binary bitstream. During decoding, a synthesis transform network is responsible for reconstructing the quantized features to produce the decoded image. When assessing image quality, this method not only employs the Peak Signal-to-Noise Ratio (PSNR) metric but also utilizes the Multi-Scale Structural Similarity (MS-SSIM) index [31]. Through end-to-end training and optimization, this approach effectively improves coding efficiency and visual quality, especially in terms of maintaining high image quality at low bitrates.

The objective of nonlinear transforms is to reduce correlation redundancy in the image space. The GDN achieves spatial decorrelation to some extent and can be embedded within networks. In order to further reduce spatial redundancy, Liu et al. [32,33] investigated the utilization of nonlocal attention mechanisms. Chen et al. [34] proposed a simplified nonlocal attention module by removing the nonlocal block and constructing an attention module based on residual blocks to capture a wider receptive field. This approach enables a more focused representation of challenging regions with fewer bits. However, the inter-channel correlations are often neglected; therefore, to emphasize the relationships between potential representation channels, Liu et al. [35] introduced a channel attention mechanism. To further exploit spatial redundancy, Akbari et al. [36] proposed an innovative dual-resolution image encoding scheme by dividing the image representation into high-resolution and low-resolution components, which significantly reduces spatial redundancy. To enhance the performance of traditional convolutions in feature extraction, Ye et al. [37] introduced an asymmetric convolution module. Ma et al. [38,39] proposed a type of wavelet-like transform that retains all information when converting images to latent representations, offering higher interpretability compared to traditional CNNs. Moreover, Xie et al. [40] presented an improved reversible encoding network that utilizes invertible neural networks (INNs) to substantially reduce information loss during the conversion between images and their latent representations. A summary of important contributions of image compression in recent years is shown in Table 1.

Since different images have distinct textures, it is necessary to apply appropriate transforms to images with varying textures to generate compact representations. To this end, Lu et al. [45] first proposed integrating convolution and self-attention units to form content-adaptive transforms, dynamically representing and embedding neighborhood information for any input. Wang et al. [41] constructed a neural data-dependent transform that enhances the functionality and flexibility of the decoder. Pan et al. [42] utilized hyper-priors to generate side information to guide the decoder in reconstructing images, with side information compensating for the loss of information during transform. In addition to reducing information loss, it is also important to exploit the local redundancy of images. Therefore, Zou et al. [43] proposed a window-based attention module to remove local redundancy in images. Since there is also global spatial redundancy in images, Ruan et al. [46] introduced a hybrid global and local context module that combines global context extractors with local context extractors in a parallel design to capture global and local dependencies. Liu et al. [44] proposed an efficient parallel Transformer–CNN hybrid module that combines the local modeling capabilities of CNNs with the global modeling capabilities of Transformers to improve the overall architecture of image compression models.

Despite the progress made in these studies, most CNN-based methods apply a uniform transform to all input images to generate a compact representation, which limits their ability to apply appropriate transforms to images with different textures. Furthermore, existing methods still struggle to simultaneously exploit the correlation between global and local features within images, resulting in an inability to effectively eliminate redundancy and constraining improvements in encoding performance.

To tackle the issues previously mentioned and create efficient image representations for a range of textures, we developed the syntax-guided content-adaptive transform model (SGCATM). We introduced the syntax-refined side information module (SRSIM) to harness syntax and side information for guiding the adaptive transforms of content features. We also designed global–local, local–global, and upsampling/downsampling modules to capture both global and local image correlations effectively. The global–local module in the encoder captures global context before focusing on local details, while the local–global module in the decoder extracts local features and then expands to include global attributes. This asymmetric design allows for a deeper understanding of image details during encoding and decoding, reducing redundancies and improving rate-distortion performance.

Our experiments show that our model adapts to the complex characteristics of various images, enhancing image compression efficiency. Tested on three public datasets, it has shown superior rate-distortion performance. This paper contributes three key points.

- We introduced the syntax-refined side information module in the decoder, which fully utilizes the syntax and side information to guide the adaptive transform of content features. This enhances the decoder’s ability to perform nonlinear transforms.
- Within the encoder and decoder, we created distinct global-to-local and local-to-global modules aimed at tapping into both global and local redundancies within images, thereby enhancing coding performance further.
- We proposed upsampling and downsampling modules to further capture the global correlations within images, thereby enhancing the coding performance of the model.

## 2. Materials and Methods

### 2.1. Datasets and Data Processing

We trained our model using the DIV2K dataset [47], and for evaluation, we utilized the Kodak dataset [48], the professional subset of the CLIC validation dataset [49], and the legacy Tecnick test set [50]. The Kodak image set consists of 24 images, all with resolutions 768 × 512. We utilized the RGB image set from the old Tecnick test set, which consists of 100 images with a resolution of 1200 × 1200. The evaluation of the CLIC validation dataset reveals the performance of the proposed method on images of higher resolutions, i.e., 1803 × 1175 on average.

During the training phase, the input images were randomly cropped into 256 × 256 patches using a batch size of 8. We utilized the Adam optimizer and conducted 500,000 iterations, starting with a learning rate of 1 × 10^−4^. The learning rate was reduced to 5 × 10^−5^ after 400,000 iterations and was further decreased to 2.5 × 10^−5^ after 450,000 iterations. When optimizing for PSNR, the trade-off parameters, i.e., λ values between bitrate and distortion, were set to {0.001, 0.0015, 0.0025, 0.008, 0.015, 0.02}, and we trained six different models for varying bitrates. For optimization targeting MS-SSIM, the λ values were set to {6, 16, 21, 64, 121}, and we trained five different models for varying bitrates.

In the testing phase, for each image, we loaded the pre-trained models mentioned above and fine-tuned the encoder using the Adam optimizer with a learning rate of 1 × 10^−5^ for 100 iterations. It is important to note that, due to the significant memory requirements for fine-tuning on high-resolution images, we only performed fine-tuning on the Kodak dataset and not on the CLIC dataset.

### 2.2. The Proposed Syntax-Guided Content-Adaptive Transform Model

**Figure 1:**
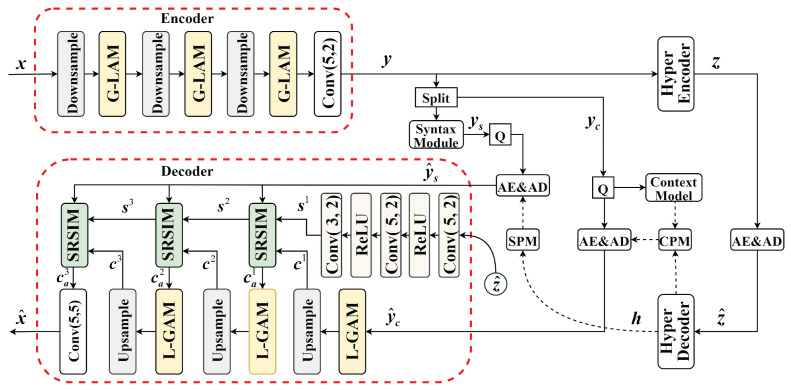
The syntax-guided content-adaptive transform model architecture.

Our proposed syntax-guided content-adaptive transform model (SGCATM) is primarily composed of an encoder, a decoder, and an entropy model. Figure 1 illustrates SGCATM architecture. The model we introduce is designed to enhance the coding abilities of both the encoder and the decoder; therefore, we have opted to provide a detailed depiction of the encoder while presenting the hyper-encoder in a more simplified form. To effectively extract global and local texture features, we propose the integration of a global–local attention module (G-LAM) within the encoder and a local–global attention module (L-GAM) within the decoder.

In the encoder, the input image *x* is passed through an encoder to transform it into a latent representation $y_{c}$. This latent representation is then divided into two parts: the portion with more channels, regarded as content $y_{c}$, is used to learn the contextual information of the image, while the portion with fewer channels is fed into the syntax module to generate syntax information $y_{s}$.

In the entropy model, a hyper-encoder is utilized to learn the hyper-prior *z* within the latent representations *y*, while a hyper-decoder is employed to learn the global structural parameters *h* of the latent representations *y*. These parameters guide the syntax probability model (SPM) and the content probability model (CPM), respectively, to estimate the probability distributions of the quantified syntax $\hat{y_{s}}$ and content $\hat{y_{c}}$. An arithmetic encoder (AE) is used to compress the quantified syntax $\hat{y_{s}}$ and content $\hat{y_{c}}$ into a binary stream, and an arithmetic decoder (AD) is used to reconstruct the content and syntax from the binary stream.

In the decoder, the decoded hyper-prior $\hat{z}$ generates side information $s^{1}$ through a series of transforms. This side information, in conjunction with decoded syntax $\hat{y_{s}}$ guidelines, facilitates the generation of content features $c_{a}^{1}$ through the SRSIM that are better suited to complex attributes, such as texture and structure. The first layer of refined side information $s^{1}$ is upsampled by a factor of two to obtain $s^{2}$ through the SRSIM. Alongside the quantified syntax, $\hat{y_{s}}$, $s^{2}$ is processed through the SRSIM, generating the second layer of refined side information $s^{2}$ and $s^{3}$. This iterative process continues, with each layer’s refined side information $s^{l}$ and decoded syntax $\hat{y_{s}}$ guiding the content $c^{l}$ through the SRSIM for feature transform to obtain $c_{a}^{l}$. This process applies to layers l=1,2,3. Ultimately, $c_{a}^{3}$ undergoes convolution to produce the final decoded image $\hat{x}$.

Additionally, in Figure 1, solid arrows represent the regular input and output, while dashed arrows indicate the Gaussian parameters, namely, the mean and variance. The $z_{h}at$ inside a circle denotes the quantified hyper-prior $z_{h}at$, equivalent to the quantified hyper-encoder output. This depiction is necessary because directly drawing a line from the hyper-encoder to the decoder would make the structural diagram aesthetically unpleasing.

### 2.3. Syntax-Refined Side Information Module

Most images possess complex attributes, and designing transforms suitable for images with different attributes can remove more spatial redundancy and achieve adaptive compression. However, existing content-adaptive transform methods are not flexible enough for decoding complex images, which results in a high degree of redundancy in the transforms. Addressing this issue, we propose the idea of using hyper-priors and neural syntax to simultaneously guide the transforms and design the SRSIM to focus more accurately on the complex attributes of the images, thereby enhancing the coding performance of the images.

**Figure 2:**
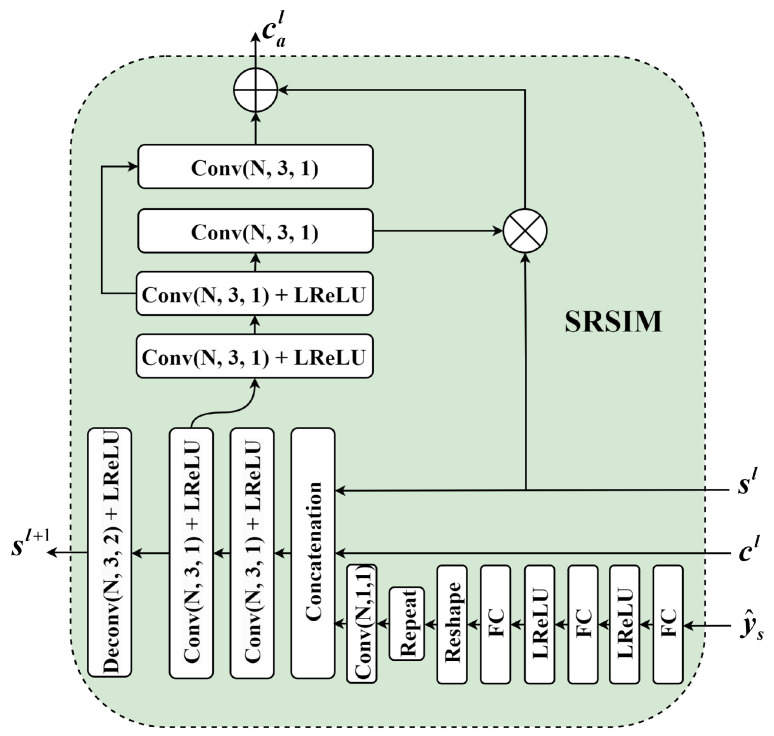
The syntax-refined side information module.

To build a transform that adapts to complex images, the proposed SRSIM is shown in Figure 2. In the initial SRSIM of the decoder, the decoded syntax $\hat{y_{s}}$ is first passed through a set of transforms and then concatenated with the content feature $c^{l}$ and side information $s^{l}$ across channels. Following this, the concatenated features are fused via two convolutional layers. The fused features are then upsampled to yield side information $s^{l+1}$. Meanwhile, the fused features are subjected to a series of spatial feature transforms to produce a content feature $c_{a}^{l}$ that is better adapted to the image properties.

### 2.4. Global–Local and Local–Global Attention Module

**Figure 3:**
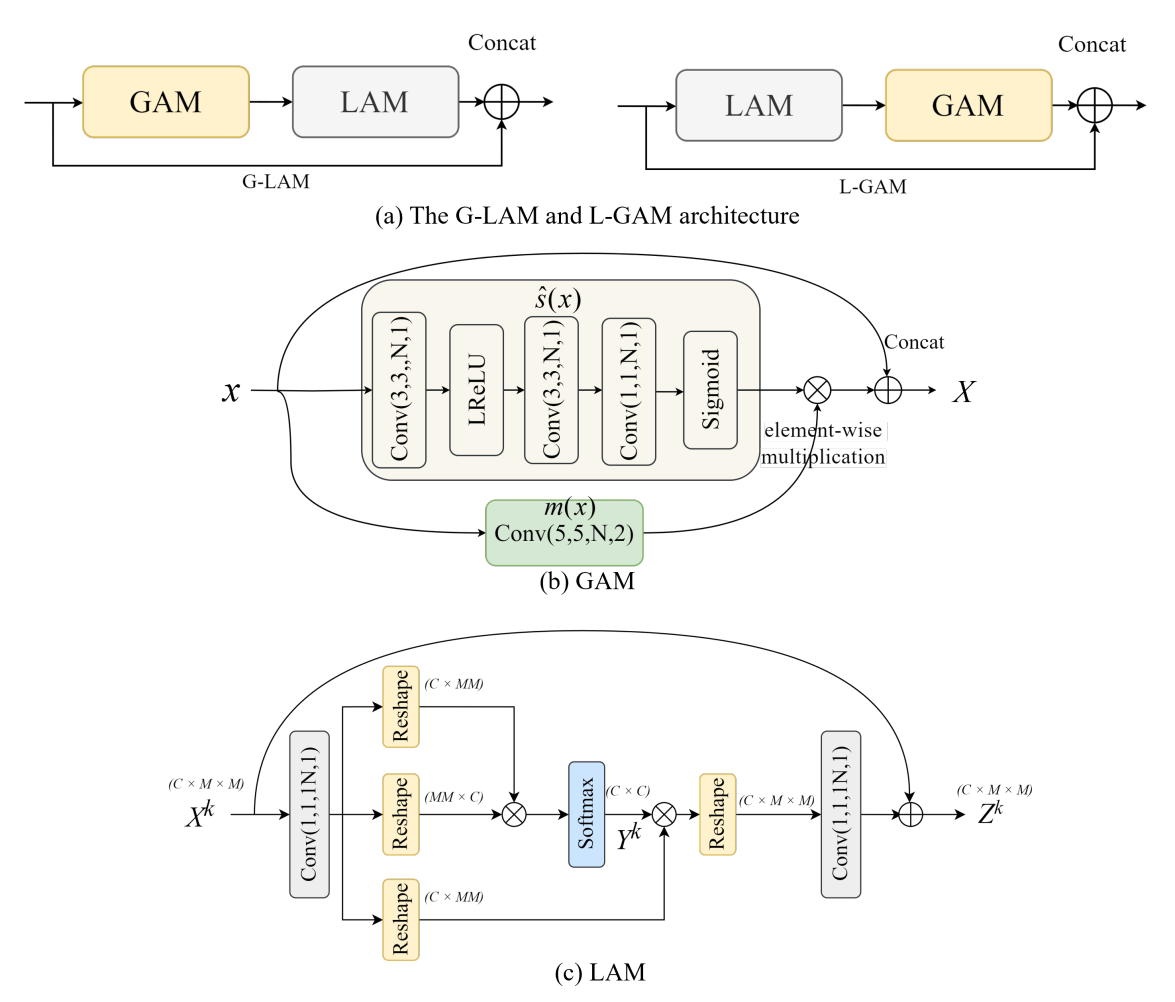
The G-LAM and L-GAM architecture.

To facilitate the extraction of both global and local textures for the purpose of augmenting rate-distortion (RD) performance, we introduce the global–local attention module (G-LAM) and the local–global attention module (L-GAM). Figure 3a illustrates the G-LAM and L-GAM diagrams. These two modules consist of a global attention module (GAM) and a local attention module (LAM).

Most current methods utilize GDN to control the input value variance of intermediate features, achieving adaptive and nonlinear adjustments along both spatial and channel dimensions. However, GDN has only a 1 × 1 receptive field, which limits its ability to learn global features. Additionally, the GDN formulation includes a square root, which results in a small degree of freedom, leading to the features scaled by GDN exhibiting low nonlinearity. To address these limitations, we propose the GAM. Figure 3b illustrates the diagram of the GAM. This module can be expressed as follows:

(1) $${\hat{s}}_{i}\left(x\right)=\frac{1}{1+e^{\beta_{i}}\cdot e^{{\left[F(x)\right]}_{i}}},$$

(2) $$X=m\left(x\right)\cdot \hat{s}\left(x\right)+x.$$

Here, *x* denotes the G-LAM module’s input; *X* denotes the output of GAM within the G-LAM; ${\hat{s}}_{i}$ denotes a scaling factor along the output channel dimensions; $\beta_{i}$ denotes a dimension of learnable parameters along the output channel dimensions; F(·) refers to a generic convolutional neural block; m(x) is the mapping of input *x*; and $\hat{s}\left(x\right)$ represents the function that determines the scaling factor. Figure 3b enables all learnable parameters to assume both negative and non-negative values, thereby enhancing the nonlinear capacity of the GAM. Furthermore, Figure 3b endows the model with a 5 × 5 receptive field and, through the inclusion of a residual structure, facilitates a more stable training process.

**Figure 4:**
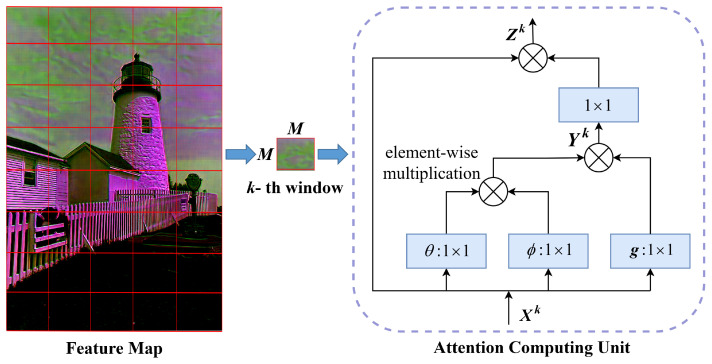
The attention masks are computed in a local window.

In image compression tasks, eliminating local redundancy is crucial for better reconstruction of the image’s local details and textures. However, most attention mechanisms focus on global information. Therefore, we introduce a novel local attention module called LAM, which is based on the Swin-Transformer attention mechanism and specifically targets local details. The structure of LAM is illustrated in Figure 3c. Here, *C* represents the number of channels; *M* indicates that the feature map in Figure 4 has been divided into windows of size M × M; C × M × M denotes the dimension of tensor $X^{k}$ as (C,M,M); C × MM indicates that, after reshaping, the dimension of this tensor has changed from three-dimensional (C,M,M) to two-dimensional (C,MM); and MM × C indicates that, after reshaping, the dimension of this tensor has changed from three-dimensional (C,M,M) to two-dimensional (MM,C).

As shown in Figure 4, to effectively compute the attention map and enhance the encoding performance, we divide the feature map into several non-overlapping windows, each with a length and width of *M*. Before the attention calculation, a 1 × 1 convolution is applied to readjust the input features. Then, the attention map within each window is computed separately. The *i*-th and *j*-th elements of the *k*-th window are denoted as $X_{i}^{k}$ and $X_{j}^{k}$, with $Y_{i}^{k}$ defined as:

(3) $$Y_{i}^{k}=\frac{1}{C(X^{k})}\sum_{\forall j}f\left(X_{i}^{k},X_{j}^{k}\right)g\left(X_{j}^{k}\right).$$

Here, $f\left(X_{i}^{k},X_{j}^{k}\right)=e^{\theta {\left(X_{i}^{k}\right)}^{T}\phi \left(X_{j}^{k}\right)}$, $g\left(X_{j}^{k}\right)=W_{g}X_{j}^{k}$, and $C\left(X^{k}\right)=\sum_{\forall j}f(X_{i}^{k},X_{j}^{k})$. The feature $X^{k}$ undergoes a series of operations, including a 1 × 1 convolution followed by a reshaping process, resulting in two matrices, θ and ϕ. Subsequently, θ is transposed, and its inner product with ϕ is computed, yielding a feature of size *C* × *C*. Assume $\theta \left(X_{i}^{k}\right)=W_{\theta }X$ and $\phi \left(X_{i}^{k}\right)=W_{\phi }X$, where $W_{\theta }$ and $W_{\phi }$ are cross-channel transforms, and $W_{g}$ is a non-cross-channel transform. The function f(·) is an embedded Gaussian function, and $C(X^{k})$ is a normalization factor. For given *i* and *k*, $\frac{1}{C(X^{k})}f\left(X_{i}^{k},X_{j}^{k}\right)$ denotes the softmax normalization calculation across the *j*-th dimension of the *k*-th window. After the attention calculation, a 1 × 1 convolution is applied to adjust the features, and finally, a residual connection is added to stabilize the training. Therefore, the output is as follows:

(4) $$Z_{i}^{k}=W_{z}Y_{i}^{k}+X_{i}^{k}.$$

Here, $W_{z}$ is a weight matrix used to compute the positional embeddings on $Y_{i}^{k}$.

### 2.5. Downsampling and Upsampling Modules

**Figure 5:**
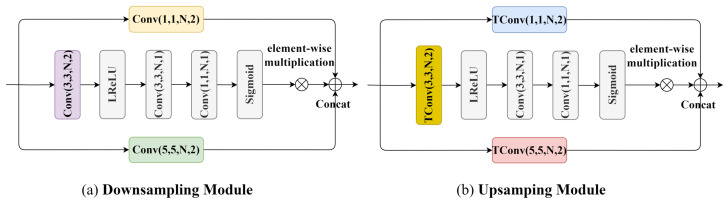
The downsampling and upsampling module architecture.

To retain more global information during feature downsampling and to utilize a greater amount of global features for image reconstruction during upsampling, we have designed downsampling and upsampling modules. The structure of these modules is similar to that of the GAM, as illustrated in Figure 5, where Tconv refers to transposed convolution. We replace the standard convolutional layers with our proposed downsampling and upsampling modules to further enhance the compression performance of the model.

### 2.6. Loss Function

The loss function for the compression model can be expressed as follows:

(5) $$L=R_{s}+R_{c}+R_{z}+\lambda \cdot D\left(x,\hat{x}\right).$$

Here, $D(x,\hat{x})$ represents the distortion between the reconstructed image $\hat{x}$ and the original image *x*. Mean Squared Error (MSE) is used as a measure of distortion in the experiments, and $R_{s}$, $R_{c}$, and $R_{z}$ represent the required bitstream for syntax, content, and hyper-priors, respectively. The Lagrange multiplier λ serves as a tuning parameter to achieve a balance between the bitrate and distortion across the entire compression system. During the training phase, actual encoding and decoding processes are omitted, and the bitrate is approximated based on the entropy of the syntax, content, and hyper-priors.

## 3. Results

### 3.1. Rate-Distortion Performance

**Figure 6:**
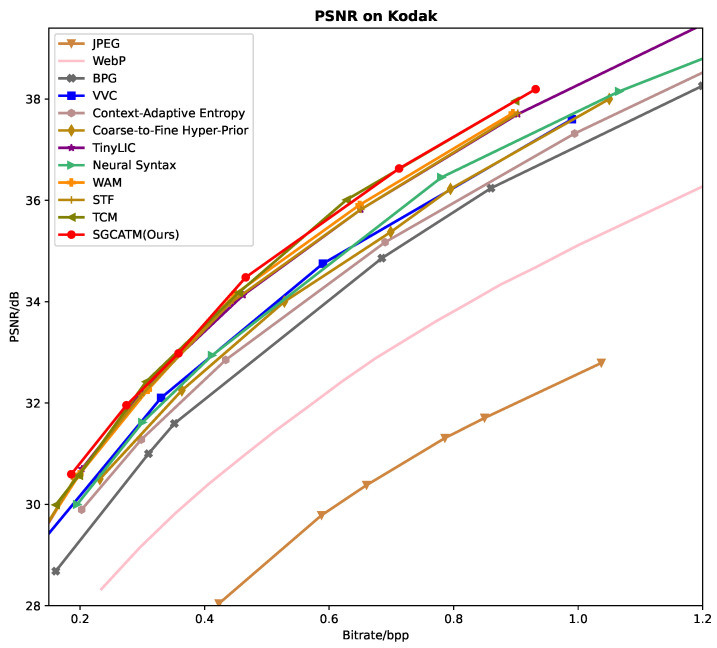
The PSNR optimized RD curve on the Kodak dataset.

**Figure 7:**
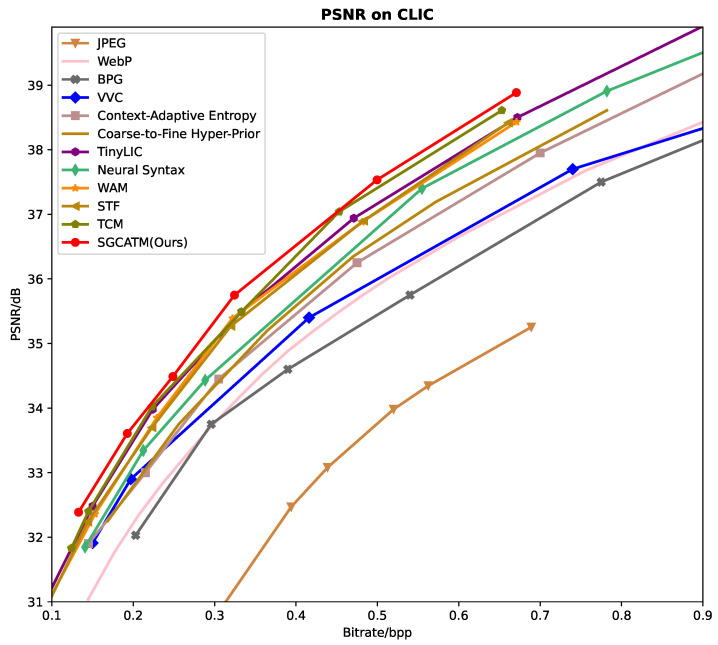
The PSNR optimized RD curve on the CLIC dataset.

**Figure 8:**
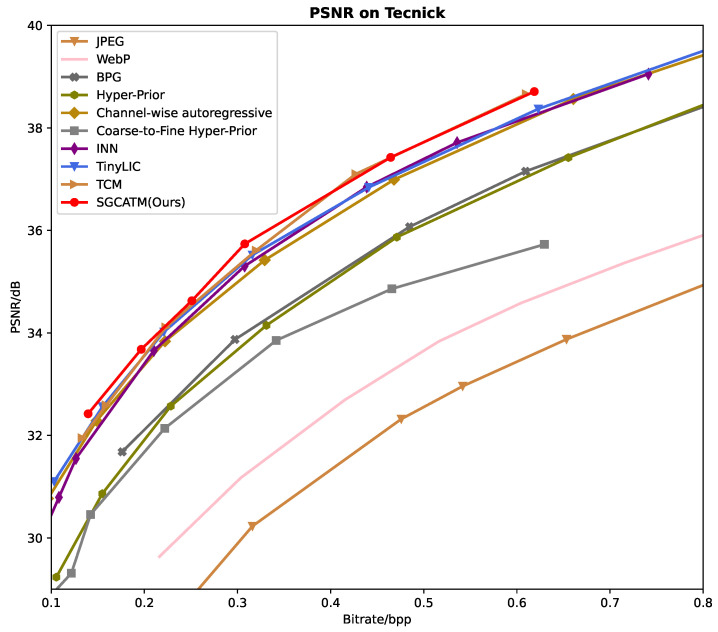
The PSNR optimized RD curve on the Tecnick dataset.

**Figure 9:**
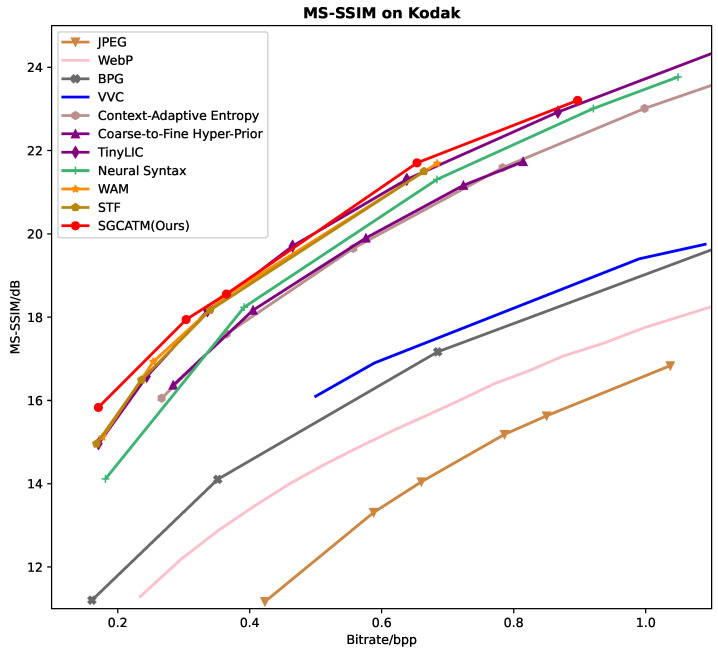
The MS-SSIM optimized RD curve on the Kodak dataset.

**Figure 10:**
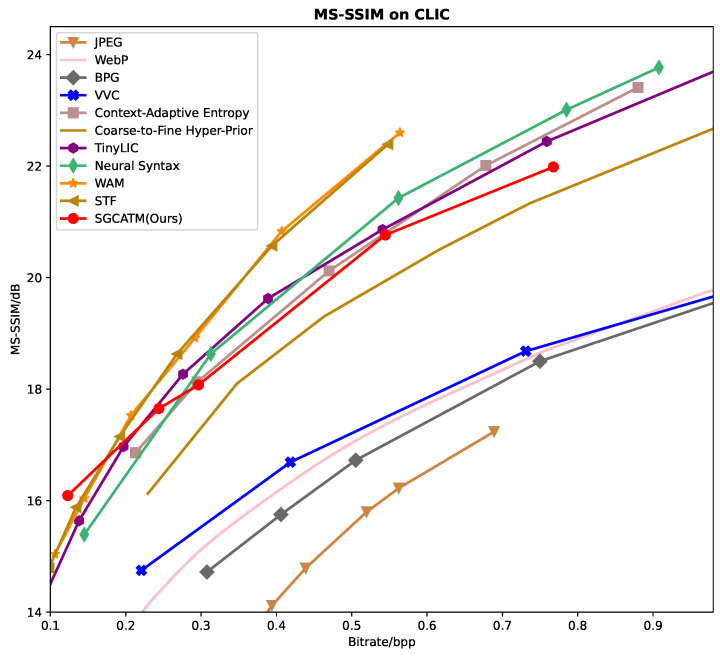
The MS-SSIM optimized RD curve on the CLIC dataset.

**Figure 11:**
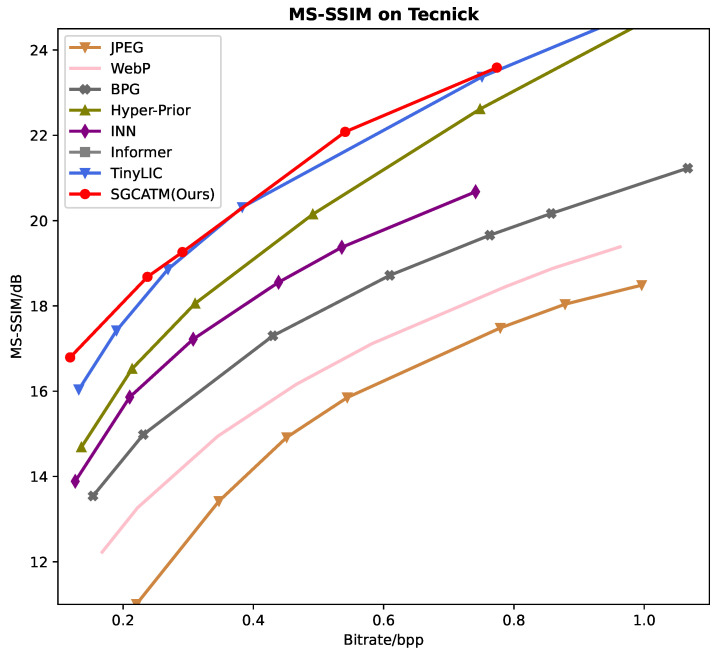
The MS-SSIM optimized RD curve on the Tecnick dataset.

Figure 6, Figure 7 and Figure 8 present the RD curves of the PSNR optimized SGCATM on the Kodak, CLIC, and Tecnick datasets, respectively. Compared with various existing traditional methods [14,16,17,18] and recent learning-based approaches [22,24,25,26,40,41,43,44,45], especially the state-of-the-art method TCM [44], the proposed SGCATM achieves the highest PSNR values under the same bitrate, demonstrating the effectiveness and superiority of the proposed method. Figure 9, Figure 10 and Figure 11 present the RD curves of the MS-SSIM optimized SGCATM on the Kodak, CLIC, and Tecnick datasets, respectively. On the Kodak and Tecnick datasets, our SGCATM achieved the highest MS-SSIM values compared to TinyLIC [45], WebP [17], BPG [16], and JPEG [14], demonstrating the effectiveness of the SGCATM. However, on the CLIC dataset, our SGCATM performed less effectively at high bitrates compared to the Neural Syntax method [41]. The reasons for the suboptimal performance may include the following. Firstly, unlike SFT [43] and WAM [43], our entropy model does not focus on the redundancy between channels. Secondly, the LAM within SGCATM is more suited to preserving texture at low bitrates. However, at high bitrates, the local attention module is less effective. Moreover, when integrated with other modules, the encoding performance may not be as good as that achieved by alternative methods.

To highlight the efficiency of the model’s performance, we also calculated the BD-rate values for different methods compared to BPG [16]. BD-rate, measured in percent (%), is an indicator used to assess the RD performance of image/video coding, serving as one of the metrics for evaluating the efficiency of image coding. During the image coding process, as the bitrate decreases, the PSNR may also decrease. In such cases, BD-rate emerges as an effective tool for measuring RD performance. A negative BD-rate calculation implies that the optimized algorithm outperforms the original one in terms of RD performance. The lower the BD-rate, the higher the compression efficiency of the algorithm, meaning that a lower bitrate is required under the same PSNR conditions.

As shown in Table 2, under the same image quality conditions, the proposed SGCATM model achieved a 25.3% bitrate saving for the Kodak dataset compared to BPG, a 38.5% bitrate saving on the CLIC dataset, and a 30.8% bitrate saving on the Tecnick dataset. Furthermore, the SGCATM model outperformed the state-of-the-art method TCM in terms of bitrate savings on the CLIC and Tecnick datasets, and its BD-rate value on the Kodak dataset is competitive with TCM.

It should be noted that the channel-wise autoregressive model’s [25] source code is not publicly available. The test data on the Kodak and Tecnick datasets presented in Table 2 were sourced from the CompressAI platform [51], which does not provide test results for the CLIC dataset; hence, we are unable to present test data for this dataset. Furthermore, the implementation of the VVC method was carried out using the VTM12.1 toolkit. Given that the Tecnick dataset comprises hundreds of high-resolution images, testing its BD-rate would require a substantial amount of time. Consequently, the VVC method has yet to conduct BD-rate testing on the Tecnick dataset, opting instead to test on another high-resolution dataset, CLIC.

### 3.2. Subjective Quality Comparisons

For a more intuitive comparison, this paper visualizes the experimental results trained on PSNR and MS-SSIM, respectively. The BPG implementation used is version bpg-0.9.8, and the VVC version is VTM12.1.

**Figure 12:**
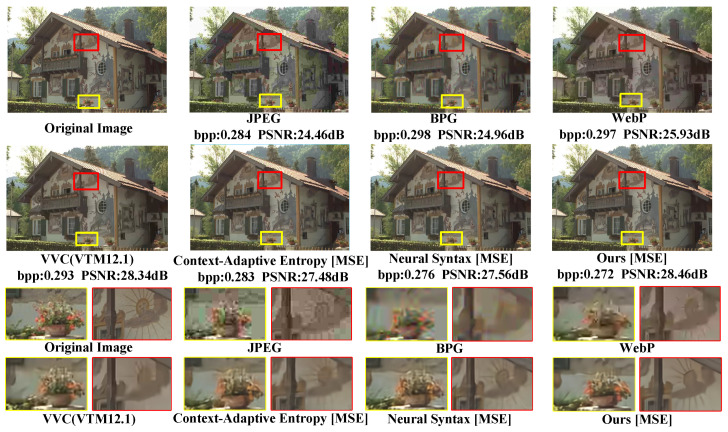
Subjective evaluation of Kodim24, which is optimized for PSNR. We compare our SGCATM with JPEG [14], BPG [16], WebP [17], VVC [18], Context-Adaptive Entropy [24], and Neural Syntax [41].

**Figure 13:**
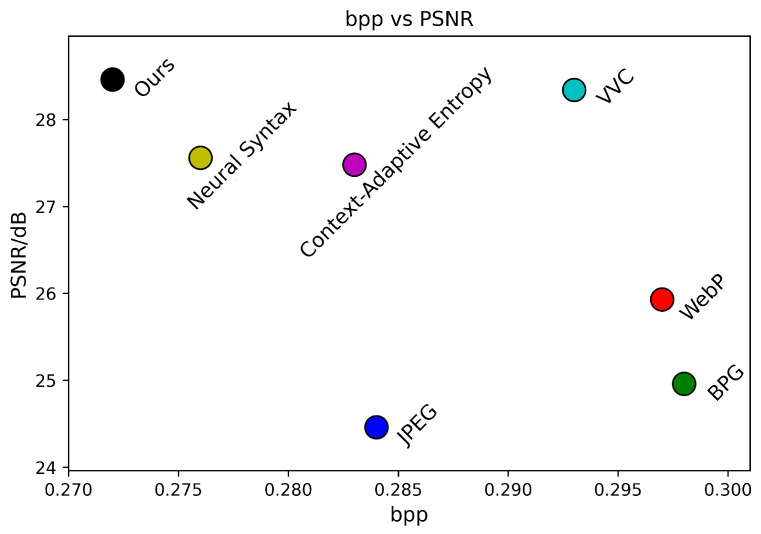
PSNR–bpp during testing on Kodim24.

The subjective evaluation of Kodim24 based on PSNR is depicted in Figure 12. To more intuitively observe the impact of different models on the compression performance of Kodim24, we have plotted the PSNR–bpp scatter diagrams for each method, as shown in Figure 13. As observed in Figure 12, none of the other methods, including the state-of-the-art traditional method VVC, have successfully reconstructed the lines in the mural. This indicates that reconstructing this area with fewer bits is highly challenging. Nevertheless, as shown in Figure 12 and Figure 13, our approach can preserve more mural details with fewer bits to obtain higher reconstructive quality compared to competing techniques, which fully demonstrates the superiority of our approach in compressing such challenging images.

**Figure 14:**
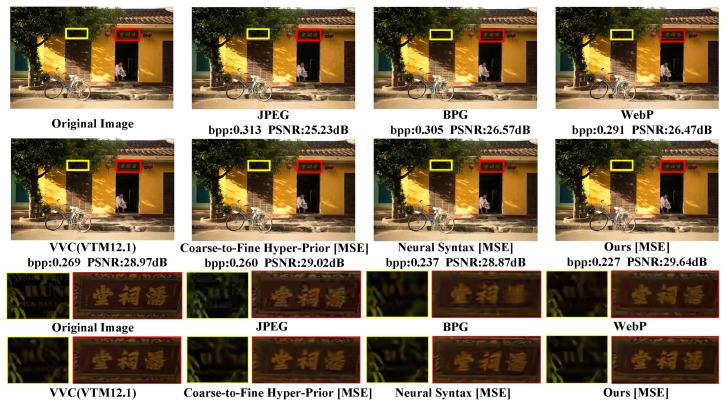
Subjective evaluation of CLIC35, which is optimized for PSNR. We compare our SGCATM with JPEG [14], BPG [16], WebP [17], VVC [18], Coarse-to-Fine Hyper-Prior [26], and Neural Syntax [41].

**Figure 15:**
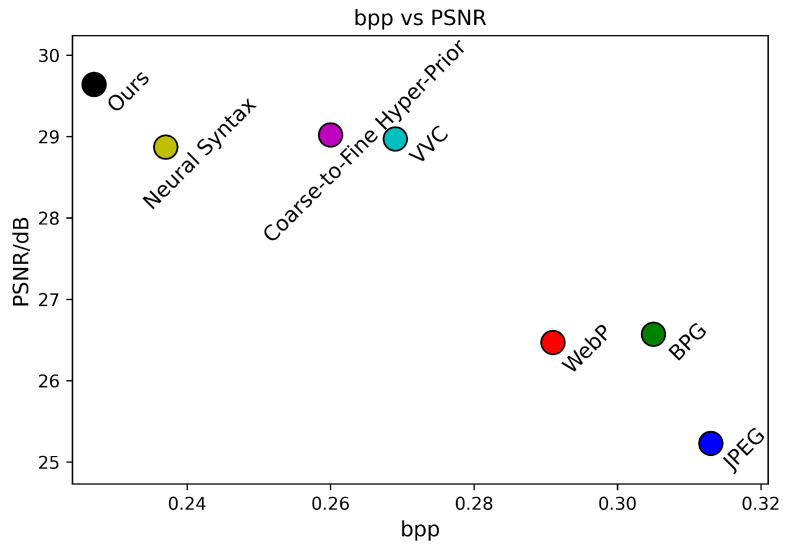
PSNR–bpp during testing on CLIC35.

We have also visualized the reconstruction results of different methods based on PSNR on the CLIC35 dataset, as demonstrated through PSNR–bpp scatter plots. In Figure 14 and Figure 15, our method retains the most text details with the lowest number of bits, achieving optimal performance. Here, ‘bpp’ denotes bits per pixel, which represents the rate.

**Figure 16:**
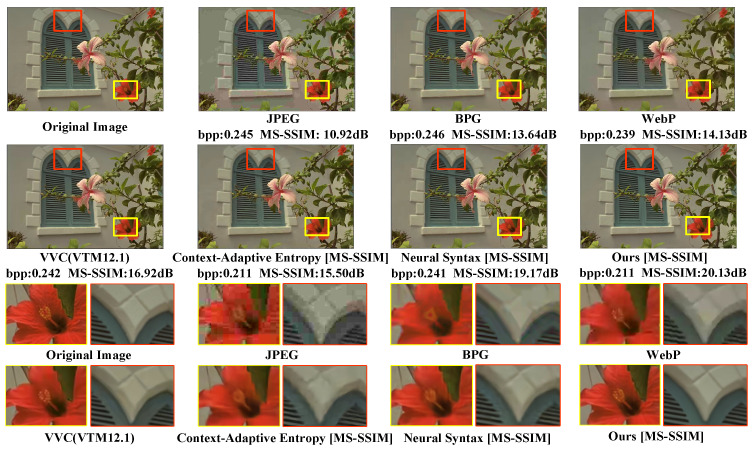
Subjective evaluation of Kodim7, which is optimized for MS-SSIM. We compare our SGCATM with JPEG [14], BPG [16], WebP [17], VVC [18], Context-Adaptive Entropy [24], and Neural Syntax [41].

**Figure 17:**
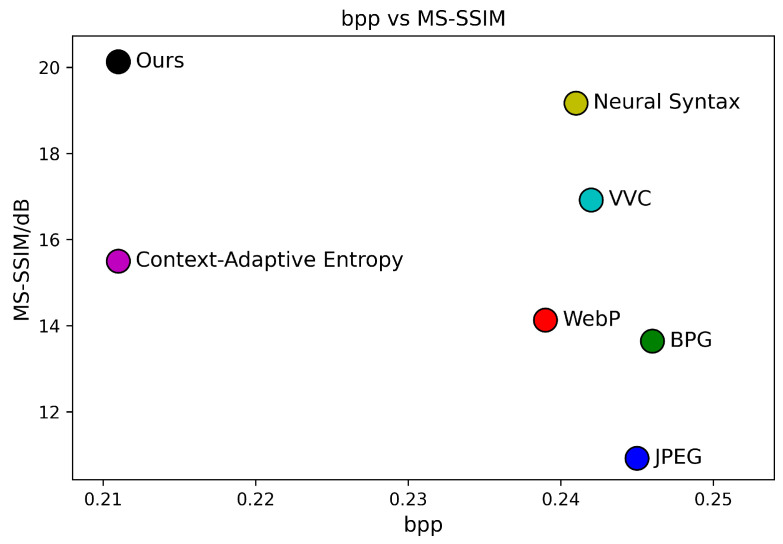
MS-SSIM–bpp during testing on Kodim7.

The subjective evaluation of Kodim7 based on MS-SSIM is depicted in Figure 16. To more intuitively observe the impact of different models on the compression performance of Kodim7, we have also plotted the MS-SSIM–bpp scatter diagrams for each method, as shown in Figure 17.

In Figure 16, our method reveals more petal patterns, pistil details, and the texture at the top of the window with greater clarity, demonstrating that the proposed method can effectively preserve the visual quality and texture details of the image while ensuring a low bitrate.

**Figure 18:**
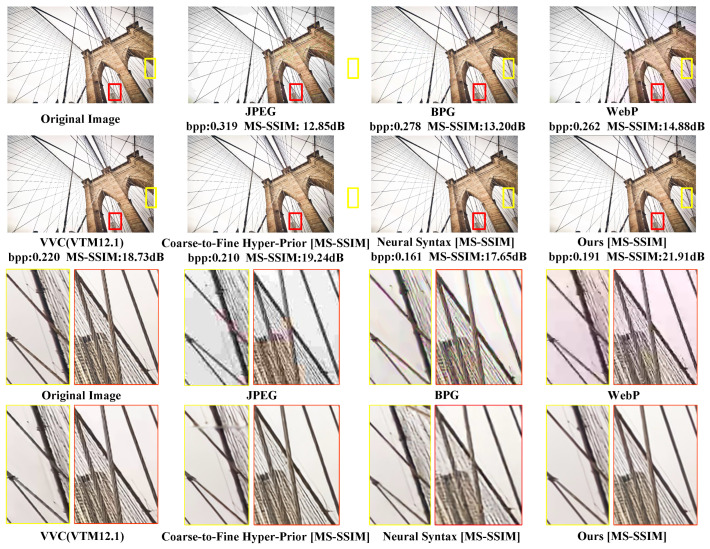
Subjective evaluation of CLIC39, which is optimized for MS-SSIM. We compare our SGCATM with JPEG [14], BPG [16], WebP [17], VVC [18], Coarse-to-Fine Hyper-Prior [26], and Neural Syntax [41].

**Figure 19:**
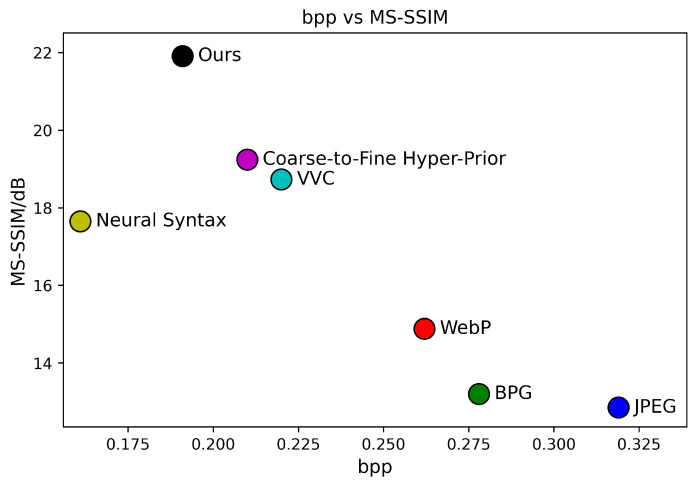
MS-SSIM–bpp during testing on CLIC39.

The subjective evaluation of CLIC39 based on MS-SSIM is depicted in Figure 18. The MS-SSIM–bpp scatter diagrams for each method are shown in Figure 19.

In Figure 18, only our method is capable of clearly reconstructing a greater number of thin ropes. Furthermore, the proposed SGCATM can still reconstruct complex texture details at a lower bitrate, exhibiting excellent coding performance.

### 3.3. Ablation Studies

**Figure 20:**
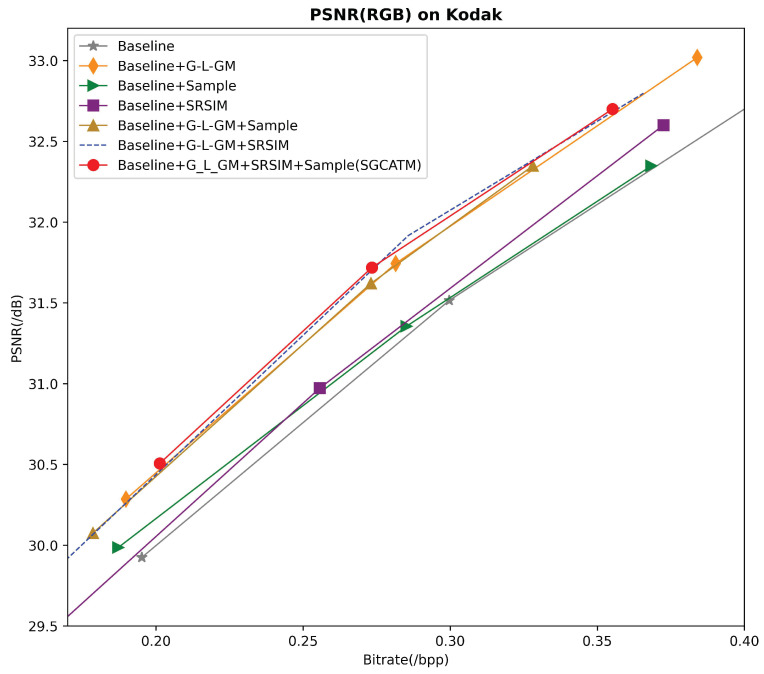
Ablation studies.

To further validate the effectiveness of the proposed SGCATM, we trained three PSNR-optimized models for each module at low bitrates and tested them on the Kodak dataset. It is important to note that, for a fair comparison, none of the models in the ablation study were fine-tuned. Initially, we named the Neural Syntax method ‘Baseline’. Then, we added the G-LAM and L-GAM to the Baseline, naming the resulting model ‘Baseline+G-L-GM’. We also incorporated the upsampling module into the Baseline, creating ‘Baseline+Sample’, and added the SRSIM to form ‘Baseline+SRSIM’. Next, we combined G-L-GAM with Sample and SRSIM separately, creating ‘Baseline+G-L-GAM+Sample’ and ‘Baseline+G-L-GAM+SRSIM’. Finally, by integrating G-L-GAM, Sample, and SRSIM, we constructed our SGCATM. The experimental results, shown in Figure 20, demonstrate that all three proposed modules contribute to performance improvements, validating the efficacy of our method.

**Figure 21:**
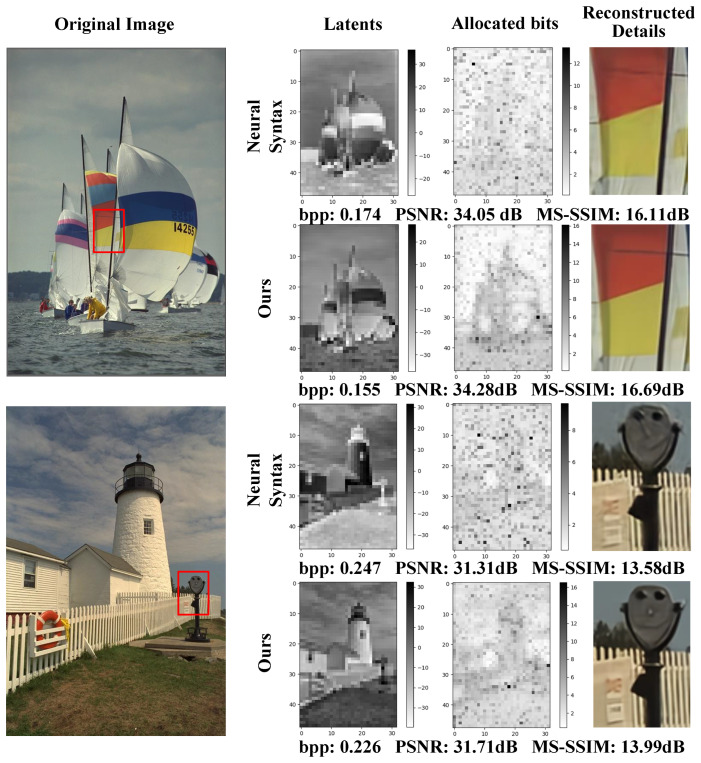
Bitrate allocation and visualization of reconstruction details of the proposed module for the channel with the latent of maximal entropy. The red frame signifies ’Reconstructed Details’.

We also focused on the entropy-maximized channels of content features in the Neural Syntax method vs. our SGCATM and conducted a visual analysis of the content features and their bitrate allocation. The specific experimental results and visualizations are presented in Figure 21. The visualization results indicate that our SGCATM concentrates on high-contrast regions (sailboats and buildings) and allocates more bits to these areas while assigning fewer bits to regions of low contrast (sky and clouds). The bitrate distribution of the Neural Syntax method is more uniform, suggesting a substantial amount of spatial redundancy in the image. Consequently, our SGCATM can better focus on the local details of the image, resulting in a more rational allocation of the bitrate.

### 3.4. Complexity Analysis

To fairly evaluate the performance of various methods, Table 3 provides parameter number and GMACs comparisons of different methods on Kodim01. The findings indicate that, although our model has a higher computational complexity, it has a smaller number of parameters. We have traded a modest increase in computational complexity for a higher performance requirement, namely, ensuring higher image quality at a lower bitrate, a contribution that is self-evident in the field of image compression.

## 4. Discussion

This paper makes a significant contribution to the field of image compression by addressing the limitations of existing content-adaptive image compression methods. The proposed SGCATM demonstrates several key advancements.

Firstly, an SRSIM is designed within the decoder. It enhances the use of syntax and side information, thereby guiding the adaptive transforms of content features. This innovation enables the decoder to perform more effective nonlinear transforms, enhancing RD performance. Secondly, the integration of global-to-local and local-to-global modules within the encoder and decoder architecture allows for a more comprehensive utilization of global and local correlations within images. Furthermore, the proposed upsampling and downsampling modules contribute to a more thorough capture of global correlations within images. By effectively modeling and leveraging these correlations, the model achieves superior rate-distortion performance, as evidenced by experimental results on three public datasets.

SGCATM innovates content-adaptive methods based on deep learning, addressing the insufficient transform capabilities of codecs and the incomplete utilization of global and local correlations. The model’s performance aligns with the trend in the field towards more complex, data-driven methods that transcend traditional compression frameworks.

When creating images with the most common color cameras equipped with a Bayer filter [52] and compressing and reconstructing these images with our model, our model demonstrates the following three main advantages:

Our model is capable of adapting to the properties and details of various color images, flexibly handling the color interpolation and spatial sampling differences introduced by the Bayer filter. It can learn to recognize and optimize these differences to maintain the color accuracy and detail of the compressed images.

The color restoration capability of our model is particularly important for processing images captured by DSLR cameras, as these images may require full-color information recovery from the original Bayer pattern. Our model optimizes the color reconstruction process by learning the relationships and transformations between the RGB channels.

For high-resolution images, our compression model maintains high quality while reducing data volume. Thanks to advanced codec technology and predictive algorithms, the model excels in compressing high-bitrate images.

Future research directions may include further refinement of SRSIM to enhance its guiding ability and exploration of other types of auxiliary information that can be integrated into the model. Additionally, extending SGCATM to other types of visual data, such as video, may be a promising direction for further enhancing compression efficiency in multimedia applications.

## Figures and Tables

**Table 1:** Summary of important contributions of image compression in recent years.

Method Name	Paper Title	Published In	Highlight
NLAIC [34]	End-to-End Learnt Image Compression via Non-Local Attention Optimization and Improved Context Modeling	TIP 2021	Embeds nonlocal network operations in the encoder–decoder and applying the attention mechanism to generate implicit masks for weighing the features of adaptive bit allocation.
Learned Bi-Resolution Image Coding [36]	Learned Bi-Resolution Image Coding using Generalized Octave Convolutions	AAAI 2021	Introduces octave convolution to decompose the latent factors into high-resolution and low-resolution components, reducing spatial redundancy.
Neural Syntax [41]	Neural Data-Dependent Transform for Learned Image Compression	CVPR 2022	This work is the first attempt to construct neural data-dependent transformation to optimize the encoding efficiency for each individual image.
CAFT [42]	Content Adaptive Latents and Decoder for Neural Image Compression	ECCV 2022	The work introduces the Content Adaptive Channel Dropping (CACD) technique, which intelligently selects the optimal quality for each part of the data and eliminates unnecessary details to avoid redundancy.
STF&WAM [43]	The Devil Is in the Details: Window-Based Attention for Image Compression	CVPR 2022	Introduces a more direct and effective window-based local attention block for capturing global structure and local texture.
TCM [44]	Learned Image Compression with Mixed Transformer–CNN Architectures	CVPR 2023	This article proposes an efficient parallel Transformer–CNN hybrid block to combine the local modeling capabilities of CNNs with the nonlocal modeling capabilities of Transformers.

**Table 2:** BD-rate results (↓) on Kodak [48], CLIC [49], and Tecnick [50]. We set our baseline as the anchor in the calculation. The best results are in **bold**, and the second-best results are underlined.

	Kodak	CLIC	Tecnick
BPG [16]	0%	0%	0%
VVC [18]	−18.1%	−13.9%	—
Channel-wise autoregressive [25]	−19.5%	—	−22.0%
Coarse-to-Fine Hyper-Prior [26]	−13.8%	−19.5%	19.0%
INN [40]	−22.1%	−29.2%	−24.7%
TinyLIC [45]	−23.9%	−33.0%	−26.0%
Neural Syntax [41]	−12.9%	−25.7%	−15.2%
WAM [43]	−23.9%	−31.9%	−27.1%
TCM [44]	**−26.1%**	−34.4%	−27.8%
SGCATM (Ours)	−25.3%	**−38.5%**	**−30.8%**

**Table 3:** Parameter number and GMACs comparison on Kodim01 of the Kodak dataset using GPU (RTX 4090). All compression methods are optimized in terms of MSE.

Method	Parameters (/M) ↓	GMACs ↓
Coarse-to-Fine Hyper-Prior [26]	74.64	713.58
STF [43]	99.86	200.6
Neural Syntax [41]	14.7	203.22
TCM [44]	45.18	212.5
SGCATM (Ours)	34.35	1296.57

## Data Availability

Data are contained within the article.
